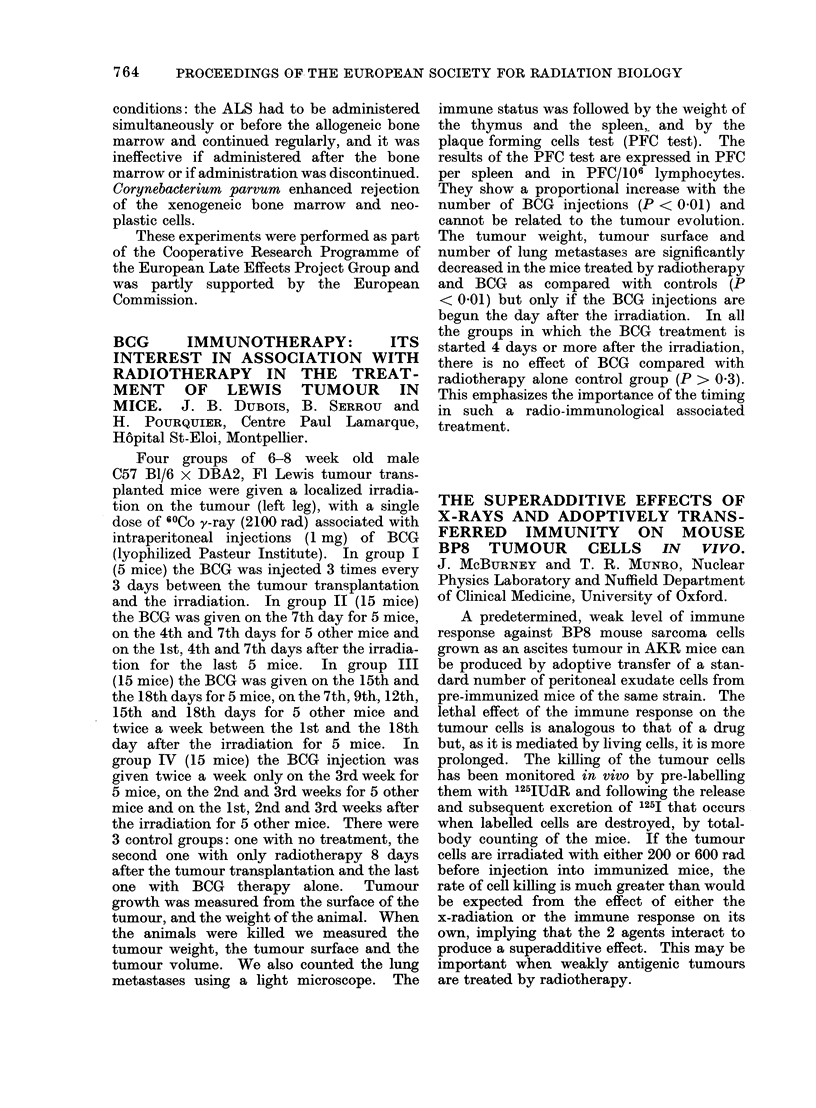# Proceedings: BCG immunotherapy: its interest in association with radiotherapy in the treatment of Lewis tumour in mice.

**DOI:** 10.1038/bjc.1975.332

**Published:** 1975-12

**Authors:** J. B. Dubois, B. Serrou, H. Pourquier


### BCG IMMUNOTHERAPY: ITS INTEREST IN ASSOCIATION WITH RADIOTHERAPY IN THE TREATMENT OF LEWIS TUMOUR IN MICE.

J. B. DUBOIS, B. SERROU and H. POURQUIER, Centre Paul Lamarque, Hôpital St-Eloi, Montpellier.

Four groups of 6–8 week old male C57 Bl/6 × DBA2, Fl Lewis tumour transplanted mice were given a localized irradiation on the tumour (left leg), with a single dose of $^{60}$Co $\gamma$-ray (2100 rad) associated with intraperitoneal injections (1 mg) of BCG (lyophilized Pasteur Institute). In group I (5 mice) the BCG was injected 3 times every 3 days between the tumour transplantation and the irradiation. In group II (15 mice) the BCG was given on the 7th day for 5 mice, on the 4th and 7th days for 5 other mice and on the 1st, 4th and 7th days after the irradiation for the last 5 mice. In group III (15 mice) the BCG was given on the 15th and the 18th days for 5 mice, on the 7th, 9th, 12th, 15th and 18th days for 5 other mice and twice a week between the 1st and the 18th day after the irradiation for 5 mice. In group IV (15 mice) the BCG injection was given twice a week only on the 3rd week for 5 mice, on the 2nd and 3rd weeks for 5 other mice and on the 1st, 2nd and 3rd weeks after the irradiation for 5 other mice. There were 3 control groups: one with no treatment, the second one with only radiotherapy 8 days after the tumour transplantation and the last one with BCG therapy alone. Tumour growth was measured from the surface of the tumour, and the weight of the animal. When the animals were killed we measured the tumour weight, the tumour surface and the tumour volume. We also counted the lung metastases using a light microscope. The immune status was followed by the weight of the thymus and the spleen, and by the plaque forming cells test (PFC test). The results of the PFC test are expressed in PFC per spleen and in PFC/$10^6$ lymphocytes. They show a proportional increase with the number of BCG injections ($P < 0{\cdot}01$) and cannot be related to the tumour evolution. The tumour weight, tumour surface and number of lung metastases are significantly decreased in the mice treated by radiotherapy and BCG as compared with controls ($P < 0{\cdot}01$) but only if the BCG injections are begun the day after the irradiation. In all the groups in which the BCG treatment is started 4 days or more after the irradiation, there is no effect of BCG compared with radiotherapy alone control group ($P > 0{\cdot}3$). This emphasizes the importance of the timing in such a radio-immunological associated treatment.